# Single-Dose Immunogenic DNA Vaccines Coding for Live-Attenuated Alpha- and Flaviviruses

**DOI:** 10.3390/v16030428

**Published:** 2024-03-10

**Authors:** Peter Pushko, Igor S. Lukashevich, Dylan M. Johnson, Irina Tretyakova

**Affiliations:** 1 Medigen, Inc., 8420 Gas House Pike Suite S, Frederick, MD 21701, USA; itretyakova@medigen-usa.com; 2 Department of Pharmacology and Toxicology, School of Medicine, Center for Predictive Medicine and Emerging Infectious Diseases, University of Louisville, 505 S Hancock St., Louisville, KY 40202, USA; igor.lukashevich@louisville.edu; 3 Department of Biotechnology & Bioengineering, Sandia National Laboratories, Livermore, CA 945501, USA; dyljohn@sandia.gov

**Keywords:** alphavirus, flavivirus, iDNA, DNA vaccine, live virus vaccine, attenuated virus

## Abstract

Single-dose, immunogenic DNA (iDNA) vaccines coding for whole live-attenuated viruses are reviewed. This platform, sometimes called immunization DNA, has been used for vaccine development for flavi- and alphaviruses. An iDNA vaccine uses plasmid DNA to launch live-attenuated virus vaccines in vitro or in vivo. When iDNA is injected into mammalian cells in vitro or in vivo, the RNA genome of an attenuated virus is transcribed, which starts replication of a defined, live-attenuated vaccine virus in cell culture or the cells of a vaccine recipient. In the latter case, an immune response to the live virus vaccine is elicited, which protects against the pathogenic virus. Unlike other nucleic acid vaccines, such as mRNA and standard DNA vaccines, iDNA vaccines elicit protection with a single dose, thus providing major improvement to epidemic preparedness. Still, iDNA vaccines retain the advantages of other nucleic acid vaccines. In summary, the iDNA platform combines the advantages of reverse genetics and DNA immunization with the high immunogenicity of live-attenuated vaccines, resulting in enhanced safety and immunogenicity. This vaccine platform has expanded the field of genetic DNA and RNA vaccines with a novel type of immunogenic DNA vaccines that encode entire live-attenuated viruses.

## 1. Introduction: Advantages of iDNA as DNA Vaccines

Here we review immunogenic DNA (iDNA) vaccines, a novel type of DNA vaccine. Recent pandemics of novel H1N1 influenza and SARS-CoV-2 emphasized the importance of novel and effective vaccine technologies. Nucleic acid vaccines such as mRNA vaccines and DNA vaccines were rapidly approved around the world to protect people from infections with SARS-CoV-2 [1]. Despite rapid progress and the approval of mRNA vaccines for SARS-CoV-2, DNA vaccines continue to raise significant interest due to the simplicity of production, genetic and thermal stability (no requirement for a cold chain), and the activation of balanced humoral, cell-mediated, and innate immunity. DNA vaccines represent one of the first nucleic acid immunization technologies [2,3,4,5]. However, despite a relatively long history and many successful preclinical trials, the clinical success of traditional DNA vaccines has been limited. Only one DNA vaccine, ZyCoV-D, has seen wide clinical use, based on its emergency use approval in India [1]. The lack of regulatory approvals is based on various factors, including relatively low DNA uptake (particularly with traditional inoculation methods), low immunogenicity in humans, and the need for repeated booster vaccinations with large quantities of DNA, as reviewed elsewhere [2,5,6,7]. Furthermore, traditional DNA and mRNA vaccines are designed to express a single vaccine-relevant antigen in the tissues of vaccine recipients, essentially serving as vectors for the transient expression of subunit antigen vaccines. Notably, subunit vaccines are known for their poor immunogenicity and often require several boosts, complex adjuvants, or inclusion into virus-like particles (VLPs) to enhance immunogenicity [8,9,10,11]. While DNA or mRNA vaccines can be manufactured rapidly, in many cases, the onset of protective immunity induced by these vaccines requires the administration of booster doses, which leads to both increased time to protection and increased manufacturing burden, ultimately resulting in additional mortality and morbidity during a rapidly developing pandemic scenario. Improvement of DNA vaccination remains an important goal for epidemic preparedness. Methods to improve DNA vaccine-induced immunity included prime-boost approaches [12,13], the development of advanced DNA vaccine delivery methods such as gene gun, microneedles, DNA injectors, or electroporation in vivo [6,14,15,16,17,18], co-expression of multiple proteins capable of forming VLPs [19], and improvements to plasmid design and manufacturing, such as addition of genes for innate immunity agonists [20,21,22,23].

**Figure 1:**
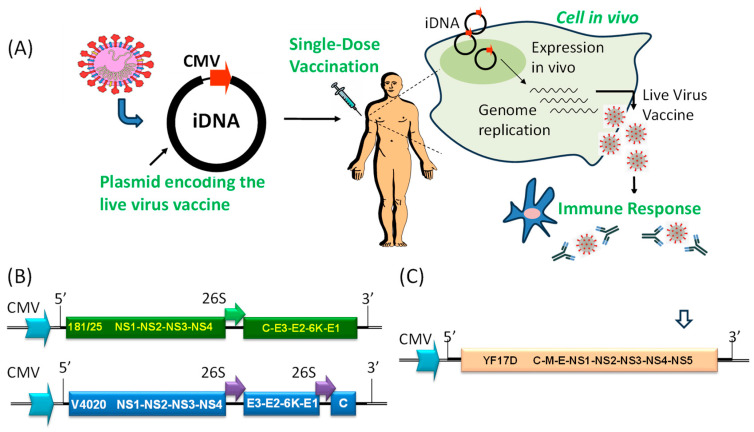
(**A**) Overview of immunization using an iDNA vaccine. The full-length viral cDNA is placed downstream from optimized eukaryotic CMV promoter sequences. In tissues injected with iDNA, transcription from the CMV promoter yields the full-length, infectious genomic RNA of a flavi- or alphavirus capable of initiating limited replication of a live-attenuated virus, thus inducing protective immune responses. For injection of DNA, syringes, microneedles, and electroporation are used [2,6,7,18]. (**B**) Genetic structures of the prototype 181/25 CHIKV iDNA vaccine (**top**), and rearranged VEEV V4020 iDNA vaccine (**bottom**). The CMV promoters and subgenomic 26S promoters, as well as regions corresponding to the 5′ and 3′ termini of genomic RNA, are shown. Proteins of CHIKV and VEEV are also shown. (**C**) Genetic structure of the YFV 17D iDNA vaccine including CMV promoter, the 5′ and 3′ termini of 17D genomic RNA, and polyproteins of YFV 17D. Approximate location of the intron is shown with an open arrow.

An iDNA vaccine platform was proposed to combine the advantages of traditional DNA immunization with the favorable immunogenicity and protective efficacy of live-attenuated vaccines [24]. The technology utilizes recombinant plasmid DNA encoding an entire genome of a live-attenuated virus. The iDNA plasmids can launch live-attenuated viruses in vitro for live-attenuated vaccine manufacturing, or directly in vivo for effective DNA vaccination [25,26,27,28,29,30,31]. These DNA vaccines represent a novel nucleic acid vaccine technology that combines the chemical and genetic stability of DNA with the exceptional efficacy of live-attenuated vaccines (Figure 1A).

To distinguish from traditional DNA vaccines, such plasmid-launched, live-attenuated vaccines sometimes were called PLLAV or iDNA vaccines because such recombinant immunogenic DNA launches replication-competent attenuated virus (vaccine), in contrast to traditional DNA immunization expressing a subunit antigen vaccine [25,26,27]. The major features of iDNA vaccines are shown in Table 1 in comparison with the traditional DNA vaccines, mRNA vaccines, and traditional live-attenuated virus vaccines.

As is any DNA vaccine, iDNA plasmids are isolated from bacteria such as *E. coli*, using an inexpensive, well-established process. Notably, DNA production can be automated and manufactured at many facilities around the world, which is important for epidemic and pandemic preparedness. All iDNA plasmids include a eukaryotic promoter, for example cytomegalovirus (CMV) promoter, which drives transcription of positive-sense viral RNA. However, unlike standard DNA vaccines that direct the transcription of mRNA for a subunit antigen, iDNA vaccines transcribe the full-length viral RNA of a live-attenuated vaccine virus. The viral RNA of positive-sense RNA viruses then starts limited replication of live-attenuated virus in the tissues of vaccine recipients, resulting in efficient vaccination (Figure 1A). In other words, iDNA plasmid turns a few cells in the tissues of vaccine recipients into the nano-scale factories for the “manufacturing” of live-attenuated virus [25,26,31].

Thus, iDNA technology represents a novel type of DNA vaccine (Table 1). With the introduction of iDNA vaccines, DNA vaccines can be divided into (i) standard DNA vaccines that express subunit antigens and (ii) iDNA vaccines that express live-attenuated viruses. Notably, traditional live-attenuated vaccines are manufactured in a few complex and highly specialized facilities, while iDNA-derived vaccines can be generated in many facilities around the world. Furthermore, iDNA vaccine is a reverse genetics system for rational vaccine design to improve vaccine characteristics, such as adaptation to emerging variants and the introduction of mutations to prevent reversion mutations to enhance safety and genetic stability. Finally, iDNA plasmids can be used as a genetically stable storage to prepare live-attenuated virus seed in vitro for use as traditional live-attenuated vaccines or, after inactivation, as inactivated virus vaccines.

## 2. Advantages of iDNA as Live Vaccines

Unlike DNA and mRNA vaccines, live-attenuated vaccines are the oldest known vaccines stemming from Edward Jenner’s pioneering experiments with cowpox virus [32]. These vaccines are the most successful and cost-effective medical interventions in the history of medicine [33]. A live-attenuated vaccine strategy was employed to eradicate smallpox (with eradication achieved in 1980) and rinderpest disease in cattle (with eradication achieved in 2011), and significantly contributed to the nearly global eradication of poliomyelitis. Live-attenuated vaccines constitute approximately half of all currently licensed vaccines. An obvious advantage of live-attenuated antiviral vaccines is the expression of multiple viral antigens in the context of attenuated viral infection. This activates innate immune responses leading to effective processing and presentation of protective antigens on MHC molecules, which efficiently stimulates cellular and humoral immunity, resulting in life-long immunity after a single-dose vaccination [33] (Table 1). Limitations of live vaccines include vulnerability to genetic reversion mutations, often due to the error-prone replication seen in RNA viruses [34,35,36]. Additionally, the preparation of classic live-attenuated variants for highly pathogenic viruses is not always feasible for biosafety reasons. The need for a cold chain and the genetic instability, biosafety, and logistics concerns of live virus manufacturing have impeded the broad use of many live-attenuated virus vaccines. Empirical attenuation methods, which were used in the past to develop successful vaccines to control polio, yellow fever, mumps, measles, Argentine hemorrhagic fever, and others are not viewed favorably in the stringent regulatory environment. For example, the live-attenuated experimental vaccine 181/25 for Chikungunya virus (CHIKV) and the TC83 vaccine for Venezuelan equine encephalitis virus (VEEV) were developed previously and found to be immunogenic and protective in clinical trials. However, both vaccines caused adverse reactions, which have been linked to genetic reversion mutations on some occasions [37,38,39]. Therefore, improved vaccines for CHIKV and VEEV are needed. Because both 181/25 and TC83 have been clinically used under Investigational New Drug (IND) protocols, they are better positioned for vaccine development against novel CHIKV and VEEV vaccines than their counterpart wild-type pathogenic viruses [25,26]. Recently, a live-attenuated CHIKV vaccine based on the La Reunion strain (LR2006-OPY1) from East/Central/South African (ECSA) lineage with the deletion of 61 amino acids in nsP3 protein was approved, which is expected to be more resistant to reversions [40].

During vaccine production, live-attenuated viruses undergo multiple cell culture passages for the preparation of vaccine virus seed stocks and manufacturing, which can result in the variation of vaccine virus populations detected by sequencing. In some cases, such as the yellow fever virus (YFV) attenuated vaccine strain, variation remains safe [41,42]. In rare cases, variation can lead to pathogenic reversion mutations. Following immunization, replication in vivo provides further opportunity for virus variation and reversion mutations to arise. Therefore, in a hypothetical scenario when a pathogenic reversion variant is found in a patient, it is not always clear if the mutation occurred in vivo after immunization, or if it originated in the process of vaccine manufacturing and was present in the vaccine before immunization. For all live-attenuated viral vaccines, including DNA and RNA viruses, it is assumed that reversion mutations may be present as a small fraction of bulk vaccine preparations (e.g., as a minor population of the live-attenuated vaccine strain quasispecies), considering what is typically a long history of virus passages. However, quality control is needed to prevent reversion mutations to ensure safety. For example, mutant poliovirus vaccine can cause an outbreak of acute flaccid paralysis (AFP). For poliovirus vaccine, the World Health Organization (WHO) developed a Standard Operating Procedure (SOP) for poliovirus (Sabin) vaccine types 1, 2, or 3 that describes variant analysis by PCR and restriction enzyme cleavage (MAPREC), followed by calculation of the percentage of revertant variants [43].

Enhancement of the genetic stability can play a role in avoiding reversion mutations and securing an attenuated genotype. In that context, *E. coli*-produced iDNA plasmids represent genetically defined molecular clones. In vivo, the vaccine virus is continuously produced by intracellular transcription using the same DNA template in host cells receiving the iDNA. Eukaryotic RNA polymerase has a ~100-fold lower error rate compared to many viral polymerases. It has been reported that mRNA molecules synthesized by RNA polymerase II contain the least number of errors (3.9 × 10^−6^ per base pair) [44]. In contrast, the error rate of SP6 RNA polymerase is 17/11.5 = 1.48 misincorporations per one genomic RNA molecule synthesized (~11,000 nt long), or 1.34 × 10^−4^ per nucleotide copied, which is comparable to the reported error rate of T7 RNA polymerase (0.5 × 10^−4^). Therefore, bottlenecking the original “production” in host cells through RNA polymerase II during iDNA vaccination inherently enhances the stability of these constructs compared to traditionally prepared live-attenuated vaccine viruses. The low mutation rate associated with iDNA technology provides an important safety advantage of iDNA for vaccines. For example, iDNA can be used as a reference molecular clone to produce master virus seeds for live or killed vaccines with improved genetic stability. Direct injection with iDNA for vaccination in vivo is designed to further limit the probability of reversions as compared to conventional virus manufacturing. Preliminary data demonstrated the genetic stability of DNA-launched virus including the attenuating mutations [26,45,46].

Following the introduction of foreign nucleic acids, including iDNA, into host cells, innate immune responses are triggered, resulting in the production of chemokine and cytokine responses. This cascade can, in turn, amplify cellular and humoral immune responses. When iDNA plasmids are used in DNA vaccination, the DNA is expected to prime these innate immune effectors prior to launching live-attenuated virus and thus enhance immunogenicity. However, more research is needed in vitro and in vivo to fully appreciate the benefits of enhanced genetic stability and the immunostimulating effects of iDNA.

Overall, iDNA plasmids offer an advantage in preparation, storage, and use, thus improving live-attenuated virus vaccines. The manufacturing of recombinant DNA in *E. coli* is a well-known technology [22,23], and DNA vaccines are simpler in production, amenable to automation processes, and less expensive logistically in transportation and storage compared to live-attenuated viruses. Therefore, iDNA technology can potentially address the major current weaknesses of live-attenuated vaccines and enhance the genetic and thermal stability, as well as the homogeneity, of initial vaccine virus seed, virus production, and vaccine deployment logistics, including storage and transportation. Importantly, iDNA vaccines still provide protective immunity with a single dose, similarly to live-attenuated vaccines [25,26,27] (Table 1).

## 3. Potential Role of iDNA Vaccines in Epidemic Preparedness

The development of vaccines for epidemic threats is vital for containing epidemics and pandemics [47,48]. Many epidemic viruses are RNA viruses such as positive-sense (+)RNA alpha- and flaviviruses [49]. Epidemic viruses can naturally evolve, or can be engineered and deliberately or accidentally released into environment. An ideal epidemic vaccine should be rapidly developed, manufactured, tested, and deployed in response to emerging or engineered pathogens, and importantly, should rapidly elicit protective immunity, preferably with a single dose [7].

Difficulties in epidemic vaccine development include challenges of working with emerging pathogens, which often are a threat to public health and are therefore regulated by the US Federal Select Agent Program. These pathogens typically require high-level biological containment (often BSL-3 and BSL-4) and require additional biosecurity precautions [47]. Furthermore, there are hurdles in conducting clinical efficacy studies, because most epidemic-relevant diseases are unpredictable and occur sporadically. Animal models are being considered as alternative way to demonstrate efficacy to support vaccine licensing. However, despite the introduction of the “Animal Rule” by the FDA in 2002, the only vaccines licensed using this regulatory pathway are BioThrax, Anthrax Vaccine Adsorbed, approved in 2015, and the enhanced version CYFENDUS (Anthrax Vaccine Absorbed, Adjuvanted), approved in 2023, both for post-exposure prophylaxis against *Bacillus anthracis*. Although the “Animal Rule” was not applied to development and approval of ERVEBO (Ebola Zaire vaccine, live, recombinant rVSV-ZEBOV), experiments in NHPs substantially contributed to the clinical development, and the “breakthrough therapy designation” and “priority review” FDA approval mechanisms were utilized [50]. Notably, in contrast to manufacturing of commercial vaccines, there is limited incentive for the pharmaceutical industry to invest in epidemic vaccines, which are intended in most cases for either rapid response to a public health emergency, or the stockpiling of a vaccine which may never be used. In the absence of epidemics or outbreaks, the market for such vaccines in the developed countries is negligible, and includes mainly medical personnel, travelers, first responders, and military and civilian personnel in the endemic areas, who are at risk of infection with the respective viruses. Therefore, due to various scientific and economic reasons, there are currently very few vaccines approved for general use for many epidemic viruses, with only rare exceptions, such as H5 influenza and smallpox virus vaccines [51,52].

Preferably, the target therapeutic profile of an epidemic vaccine should be able to elicit protective immunity with a single dose, and the vaccine should be not sensitive to pre-existing immunity to the vaccine vector components. The iDNA vaccine can potentially provide a solution to challenges with epidemic vaccination due to several advantages: (i) rapid production facilitated by automation, (ii) local manufacturing, (iii) genetic stability, (iv) thermostability, (v) intrinsic activation of innate immunity, and (vi) the ability to elicit protection with a single dose. As does bacterially produced plasmid, iDNA contains CpG motifs and is expected to stimulate innate immunity and to effectively prime adaptive immunity [53,54]. The presence of DNA in the cytoplasm of mammalian cells is perceived as an alert signal by cGAS and other STING-dependent sensors [55,56]. In response, the immune system initiates transcription of antiviral genes such as type I interferons and the production of inflammatory cytokines such as IL-1β. Activation of innate immunity and the induction of cytokine response serves as efficient priming for the acquired virus-specific immune responses, and as a result, improvement of immune responses [56]. Small-molecule inhibitors were proposed to modulate these innate immune responses to the DNA component of DNA-launched attenuated vaccines [57]. Once replication of the live-attenuated virus is launched, pattern recognition receptors and antiviral factor activation plays an additional role in further bolstering these innate immune responses.

DNA-launched vaccines can be useful for vaccination of personnel at risk of infection with emerging and re-emerging viruses, such as hospital personnel, first responders, military service personnel, and laboratory workers. Healthcare staff need vaccinations due to their proximity to potentially infected patients [58]. The world is increasingly exposed to disease transmission risk through business and humanitarian travel between countries and continents. There are 22 million workers in the U.S. health care industry (14% of all U.S. workers), which are at risk of exposure to, and consequently, spreading non-endemic viruses in the U.S.

One-dose vaccination with iDNA vaccines may provide protection for at-risk healthy personnel from emerging alpha- and flavivirus pathogens [25,26]. As safety and efficacy are confirmed in at-risk individuals, application of iDNA vaccines can be expanded to additional population groups, including endemic regions, as well as the young and the elderly. Generally, live-attenuated vaccines are used in many groups of patients. For example, FluMist live-attenuated influenza vaccine is used in healthy individuals aged 2–49 years with no serious adverse reactions [59].

Nucleic acid vaccines, such as mRNA or DNA vaccines that deliver subunit vaccines, typically require large doses and multiple boosts to achieve protection. A single, small dose of iDNA plasmid encoding live virus vaccine can have a safety advantage as compared to approaches involving multiple boosts and containing potentially harmful excipients. Traditional vaccines often contain contaminants and excipients from manufacturing processes, which can cause adverse reactions in some individuals [60,61,62]. Reactogenicity with local reactions potentially arising from large doses have been described for mRNA and standard DNA vaccines [62,63].

Therefore, iDNA technology is positioned to address many challenges associated with emergency vaccine development and deployment. Manufacturing of iDNA requires standard methods of plasmid DNA isolation. The plasmid can be either rapidly prepared at many locations in the case of an outbreak, or stockpiled for emergency use. The thermostability of DNA has advantages for stockpiling and/or shipment than many other vaccines. DNA has a favorable temperature stability to be formulated for storage and transportation at ambient temperatures. Many iDNA vaccines described so far are derived from live-attenuated virus strains, which are not considered select agents. The safety of these parent strains has been confirmed in the clinical trials or the long history of vaccination in people (for example, yellow fever 17D vaccine). The clinical history of the parent vaccines facilitates work with these iDNA vaccines and their attenuated variants. Furthermore, iDNA vaccines are not infectious as such; they can only initiate virus replication after introduction into the nucleus by in vivo delivery methods. This contributes to the improved safety, biosecurity, and manufacturing of iDNA vaccine compared to live-attenuated virus vaccines. As a reverse genetics technology, iDNA plasmids can be quickly modified to add mutations or other changes to improve safety and immunogenicity, or to adapt vaccines to emerging biothreats and vaccine-resistant variants. The summary of published iDNA vaccines is shown in Table 2.

## 4. Immunogenic iDNA Vaccines for Alphaviruses

Alphavirus iDNA vaccines include candidate iDNA vaccines for CHIKV and VEEV, both based on the clinically validated prototype vaccines (Table 2).

### 4.1. CHIKV iDNA Vaccines

CHIKV causes outbreaks of chikungunya fever worldwide including the U.S. [68,69,70,71]. The virus belongs to the *Togaviridae* family, which also contains several other mosquito-borne arboviruses [68,69,72]. CHIKV is transmitted to people by *Aedes aegypti* and *A. albopictus* mosquitoes [73,74,75] and causes severe arthralgia, cardiovascular disease, respiratory failure, hepatitis, and central nervous system symptoms, mostly in the elderly and the young [76,77]. CHIKV is found in approximately 40 countries, which reported epidemic or endemic CHIKV, mostly in warm climate in Asia, Africa, and recently in Europe and the Americas [78]. Epidemics of CHIKV took place in the 2005–2006 in La Reunion islands in the Indian Ocean, which caused 284 deaths, and in India, with an estimated 1.3 million people affected [79,80], and there were recent outbreaks in Africa, the Caribbean, and South America. With an increase in global commerce and travel, the risk for spreading CHIKV has increased [70,71,81,82,83]. Climate changes and urbanization also favor the geographical expansion of CHIKV [84,85,86]. Traveler-associated cases have also been registered in Europe, Australia, and the U.S., and some travelers were viremic, which creates the potential to initiate infectious cycles in their home countries [87]. Given the broad, worldwide distribution of *A. aegypti* and *A. albopictus*, there is a real risk of establishing CHIKV endemicity outside of the traditional geographic areas [88]. Considering its significant public health impact, CHIKV was designated a Category C Priority Pathogen by the U.S. National Institute of Allergy and Infectious Diseases (NIAID).

Recently, the approval of a live-attenuated CHIKV VLA1533 IXCHIQ^®^ vaccine of ECSA lineage origin, with deleted 61 aa in the nsP3, suggests that live-attenuated CHIKV vaccines meet stringent safety standards. Other candidate vaccines have entered clinical phase II trials, a VLP-based vaccine and a live measles-vectored CHIKV vaccine [72,89]. Live-attenuated experimental vaccines prepared using reverse genetics have also been described [90,91]. A legacy 181/25 vaccine tested in PhaseI/II clinical trials [92] was developed by the U.S. Army from the Asian lineage strain AF15561 isolated during the 1962 outbreak in Thailand [93]. Nucleic acid vaccines, including synthetic DNA plasmids delivering protein subunits, are in development [94]. A therapeutic approach using DNA to launch a monoclonal antibody capable of neutralizing the CHIKV was also reported [95].

Experimental CHIKV iDNA vaccines were prepared and evaluated in vitro and in mice [25,46,96]. In one study, CHIKV vaccine candidates were attenuated by deleting a large part of the gene encoding nsP3 or the entire gene encoding 6K and were administered as viral particles or infectious genomes launched by DNA [96]. The resulting attenuated mutants were genetically stable, elicited neutralizing antibodies and T cell responses after a single-dose immunization, and protected C57BL/6 mice from viremia and joint swelling following challenge. Other iDNA vaccines included a construct derived from the 181/25 live-attenuated vaccine, as well as vaccines that contained additional mutations to improve safety [25,97]. For illustration, the iDNA plasmid encoding the full-length infectious genome of live-attenuated CHIKV 181/25 downstream from a eukaryotic promoter can be used to generate a typical gene arrangement (Figure 1B). Transfection with as little as 10 ng of iDNA was sufficient to initiate replication of vaccine virus in vitro. BALB/c mice vaccinated by injection-electroporation with a single 10 μg dose of CHIKV iDNA seroconverted, developed neutralizing antibody, and resisted experimental challenge with pathogenic CHIKV (Table 2). Thus, live-attenuated CHIKV vaccine can be launched in vitro or in vivo by using iDNA and represents a novel vaccination strategy [25,46].

The CHIKV iDNA plasmid represents a reverse genetics system for additional rational vaccine design. Recently, an engineered experimental iDNA vaccine that encodes a rearranged genome with the capsid gene downstream from the glycoprotein genes controlled by the subgenomic promoter, CHIKV V5040, was described [97]. Attenuating mutations derived from the CHIKV 181/25 vaccine were also engineered into the E2 gene of V5040, further enhancing its safety profile. To generate V5040 live-attenuated virus, a copy of the rearranged CHIKV genomic RNA with the attenuating mutations was cloned into the iDNA plasmid pMG5040 downstream from the CMV promoter. After transfection in vitro, pMG5040 launched replication of V5040. V5040 virus was tested in experimental murine models for safety and immunogenicity. Vaccination with a single dose of V5040 virus subcutaneously resulted in high titer of CHIKV virus-neutralizing antibodies. These data warrant further development of V5040 as a novel live-attenuated vaccine for CHIKV.

### 4.2. VEEV iDNA Vaccines

VEEV is an alphavirus that shares many structural and genetic characteristics with CHIKV. It is a NIAID Category B human and veterinary pathogen infecting humans and equids using many mosquito vectors, including *Culex, Psorophora, Mansonia* and *Aedes* species [98]. The population of these mosquitoes already exists in the U.S. [99,100,101,102]. VEEV causes epizootics and human infections in North, Central, and South America, including a 1971 outbreak in Texas [103,104,105]. Climate, ecological changes, commerce, and international travel increase the risk of virus re-emergence [69,101,106]. The virus can also be prepared in larger quantities and spread by aerosol as a biological weapon [106,107]. VEEV symptoms are initially similar to influenza and are difficult to diagnose [107]. The potentially life-threatening encephalitic effects of VEEV demand an effective VEEV vaccine [108,109]. An experimental live-attenuated vaccine, TC83, was developed in the 1960s [110] and protects from many epizootic viruses in the VEEV complex [111], such as IAB, IC and IE. In the TC83 vaccine virus, two key mutations are associated with attenuation: nucleotide 3 in the 5′ untranslated region, and amino acid 120 within E2 glycoprotein [110]. TC83 was used under an IND protocol for the vaccination of at-risk medical personnel [106,112,113]. In these clinical trials, TC83 caused adverse reactions including headache and fever in ~23% of vaccine recipients, while another ~18% of recipients did not develop necessary neutralizing antibody titers [114]. Reversion mutations are thought to be associated with adverse effects in TC83 vaccine recipients [38,108]. However, due to its long history of clinical use, TC83 represents a good starting point for proof-of-concept studies as well as for the development of improved vaccines for VEEV [38].

Examples of DNA-launched VEEV-based genetic constructs included self-amplifying RNA (sa-RNA) replicon vectors for the expression of various genes [54,115,116]. These replicon vectors represent genomic sa-RNA, in which the structural polyprotein is replaced with a heterologous gene of interest. Therefore, self-amplifying replicon RNA cannot launch live VEEV because it does not encode its structural proteins. However, sa-RNA replicons have been used as vaccine vectors for immunization against cancer [117,118] and infectious agents including epidemic-relevant viruses [119,120,121,122,123]. In DNA-launched sa-RNA replicon vectors, VEEV replicon RNA was placed under the transcriptional control of an RNA polymerase II promoter, such as CMV, resulting in transcription and subsequent self-amplification of the sa-RNA vector with enhanced expression of the gene of interest. In this design, the CMV promoter drives expression of the sa-RNA vaccine. For example, DNA-launched sa-RNA expressed 3- to 15-fold more green fluorescent protein in vitro than a traditional DNA vaccine expressing standard mRNA [54]. Injection of mice with a DNA-launched sa-RNA encoding human immunodeficiency virus type 1 gp160 enhanced humoral responses considerably compared to an equivalent dose of a traditional DNA vaccine. These enhanced immune responses were also detected at 10- and 100-fold-lower doses of the sa-RNA replicon vaccine construct [54]. With the introduction of mRNA vaccines for SARS-CoV-2 prevention, there is renewed interest in sa-RNA vaccines as well. Recently, the first sa-mRNA SARS-CoV-2 vaccine was approved following Phase 3 clinical trial [124,125]. Studies with DNA-launched VEEV replicons have paved the way for launching the full-length genomic VEEV TC83 RNA in vitro and in vivo [26,126,127,128].

An iDNA vaccine coding for live-attenuated VEEV was prepared [26] using the backbone of the live-attenuated experimental TC83 vaccine [129]. TC83 antigen was detected in the cytoplasm of iDNA-transfected CHO cells. By 48 h, all cells became positive for TC83 antigens, suggesting virus replication. The pTC83 iDNA plasmid served as a template for transcription of viral RNA in vivo, and <10 ng was sufficient to initiate replication of a genetically defined, TC83-like vaccine virus (Table 2). All BALB/c mice vaccinated i.m. with a single dose of pTC83 iDNA using electroporation have seroconverted with no adverse effects. Four weeks after vaccination, iDNA-vaccinated mice were challenged with a lethal epidemic strain of VEEV and were protected from lethal disease [26]. Five out of ten challenged animals did not have any detectable viremia after challenge, while the remaining five animals had low viremia. In contrast to iDNA-vaccinated mice, unvaccinated controls lost significant body weight, developed high viremia, and died by day 7 after challenge [26].

The synthesis of the live-attenuated TC83 virus was also observed in BALB/c mice in another in vivo transfection experiment with pTC83 iDNA. Initially, viremia was not detected by direct plaque assay; however, it was detected in plasma samples after virus amplification in Vero cells. RNA was isolated from the recovered virus, and a TC83 cDNA fragment nt 8559–9850 spanning the E2 gene was prepared by RT-PCR and sequenced, which confirmed the presence of the attenuating E2-120 mutation in DNA-launched vaccine virus [26,110].

**Figure 2:**
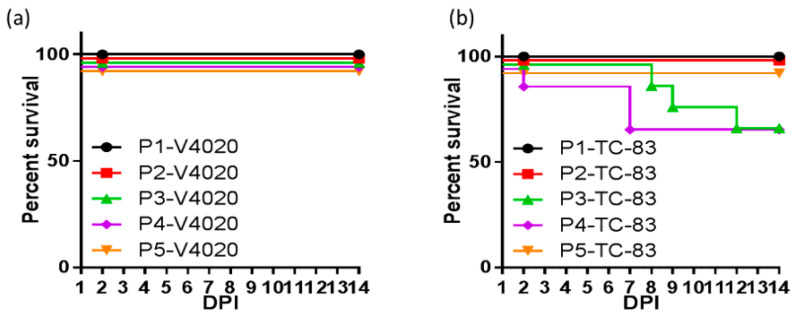
BALB/c mice (n = 10/group) were inoculated via the intracranial route with 1 × 10^5^ PFU of either V4020 or TC-83. Two days after inoculation, two mice from each group were sacrificed, brains were collected, homogenized, and used to prepare clarified viral stocks for inoculation of a subsequent group. This process was repeated four times for a total of five passages, including the initial inoculation (P1 to P5 passage number and virus is either V4020 or TC-83). Survival of (**a**) V4020 and (**b**) TC-83 passage groups was tracked during this time. Adapted from [126].

Additional iDNA VEE vaccine candidates were developed, including V4020 with a gene rearrangement and duplicated subgenomic 26S promoter, similar to CHIKV V5040 (Figure 1B). This strategy of gene rearrangement has an attenuating effect for many viruses [130,131,132]. Advantageous safety, immunogenic, and protective features of rearranged iDNA-derived V4020 VEE vaccine were demonstrated in animal models. In serial intracranial passages in mice, a standard test to assess the phenotypic and genetic stability of alphavirus attenuation, V4020 had significantly lower rates of mutations throughout five sequential passages (P1–P5) compared to the TC83 experimental vaccine (Figure 2) [126]. This genetic stability was linked with attenuation since all V4020-inoculated mice survived, while 38% of mice inoculated with TC83, P2, and P3 died.

In rabbits, a VEEV iDNA vaccine was found immunogenic using direct administration via hollow microneedles without electroporation [64]. Furthermore, V4020 vaccination of NHPs confirmed its resistance to genetic reversion and protected non-human primates from viremia after aerosol challenge [127].

## 5. Immunogenic iDNA Vaccines for Flaviviruses

DNA-launched candidate flavivirus vaccines included candidate vaccines for West Nile virus (WNV), Japanese encephalitis virus (JEV), dengue (DENV), Zika (ZIKV), and yellow fever virus (YFV) (Table 2).

### 5.1. WNV Vaccines

WNV was first discovered in 1937 in the West Nile district of Uganda and has been subsequently classified within the genus Orthoflavivirus in the family Flaviviridae. Other members of Orthoflavivirus include JEV in Asia, as well as St. Louis encephalitis virus in the New World, and Murray Valley virus in Australia, among others. Historically, circulation of WNV was observed in Africa and Asia, with rare cases in southern Europe, likely introduced by migrating birds. The emergence of this African-origin virus in New York in 1999 was an unexpected epidemic threat [133]. Currently, WNV remains an important epidemiological concern worldwide. In the U.S., *Culex* mosquitos are the primary vector that transmits the virus in the natural sylvatic transmission cycle and in the enzootic cycle to humans [134].

After WNV infection, fever develops in approximately 25% of individuals. The disease varies in clinical severity, and symptoms may last for a long time. Neuroinvasive disease, with neurological symptoms such as encephalitis, meningitis, and acute flaccid paralysis, develops in approximately 1% of infected individuals and carries a case fatality rate of ~10%. Encephalitis is associated with highly variable clinical outcomes and is often associated with long-term morbidity. A safe, efficacious, and cost-effective vaccine is needed [135]. Currently, WNV is the leading cause of mosquito-borne disease in the continental U.S. [134].

Experimental DNA-launched WNV vaccine constructs have been described [28,65] (Table 2). One construct targeting Kunjin virus, the indigenous WNV strain in Australia, was prepared with plasmid DNA directing transcription of the infectious full-length RNA genome under control of a mammalian expression promoter, and incorporating an attenuating NS1 protein Pro-250 to Leu mutation [136]. Mice vaccinated with the plasmid via the i.m. route became viremic with Kunjin virus, which was isolated from the plasma 3–4 days after DNA injection, confirming viral replication in vivo. No clinical signs of disease were observed. Neutralizing antibody was detected in serum by 19 days post vaccination. Virus challenge with varying lethal doses of virulent WNV NY99 strain or Kunjin strain i.c. or i.p, demonstrated that 0.1–1 μg of plasmid DNA protected mice from disease. These results confirmed the feasibility of delivery of an attenuated, replicating WNV via plasmid DNA as an effective vaccination strategy for WNV.

A live-attenuated WNV vaccine is not currently available. Examples of DNA-launched experimental WNV vaccine approaches were reported [30,65,66]. In the absence of a clinically approved WNV vaccine, chimeric live-attenuated virus, W1806, was prepared to attenuate the virulent NY99 strain of WNV by using mutations similar to the attenuated JEV vaccine SA14-14-2. The W1806 virus displayed promising characteristics and strong attenuation in initial experiments; however, additional attenuating mutations are needed to further improve the safety of the W1806 virus before progression to clinical trials [66].

### 5.2. iDNA Vaccine for JEV

JEV is transmitted to humans by the mosquito *Culex tritaeniorhynchus*. The virus causes epidemics throughout Asia and is the leading cause of vaccine-preventable encephalitis in Asia and the western Pacific [137]. For the past decades, killed virus vaccines prepared in tissue culture or in mouse brain passages have been used to immunize travelers to, and populations of, enzootic countries. Safety, efficacy, and cost concerns of these vaccines have led to the development of the live-attenuated vaccines SA14-14-2 and chimeric YF-JEV, as well as purified inactivated, and tissue culture-derived, vaccines [138]. An inactivated, Vero cell culture-derived, two-dose vaccine is the only JEV vaccine licensed in the U.S. [139].

Replication of DNA-launched JEV in vitro has been reported [29,67]. A JEV infectious clone was prepared as a stable DNA-launched virus, and JEV replication was demonstrated in cell culture (Table 2). To improve plasmid stability during growth in *E. coli*, this construct incorporated two introns into the JEV cDNA to disrupt a single open reading frame, which hypothetically produces proteins that are toxic in *E. coli* during plasmid propagation.

Another construct based on plasmid DNA encoding a synthetic cDNA copy of JEV SA14-14-2 live-attenuated virus under transcriptional control of the CMV promoter launched the JEV vaccine in vitro and in vivo [140]. The stability and production yields of this plasmid in *E. coli* were improved by inserting three synthetic introns into the JEV cDNA to disrupt its structural and nonstructural genes. Transfection of Vero cells with this iDNA plasmid resulted in the replication of JEV vaccine virus with intron sequences removed from viral RNA. A single-dose immunization of BALB/c mice with 0.5–5 μg of plasmid resulted in seroconversion and elicitation of JEV virus-neutralizing serum antibodies. The results confirmed the feasibility of using DNA vaccination to launch a live-attenuated JEV vaccine and support the development of iDNA-launched live-attenuated JEV vaccine.

### 5.3. ZIKV Vaccine

Tsetsarkin et al. in 2016 described an infectious cDNA clone derived from ZIKV isolated during the 2015 epidemic in Brazil [141]. Like the JEV iDNA strategy described above, to stabilize the infectious clone and to reduce plasmid toxicity during propagation of cDNA in *E. coli* (strain MC1061), two introns were introduced into NS1 (nt 2711) and NS5 (nt 8882) genes. The yield of the resulting ZIKV-ICD plasmid in *E. coli* increased by ~50%.

### 5.4. YFV iDNA Vaccine

YFV is transmitted to humans by the mosquito *Aedes aegypti* and it causes acute hemorrhagic fever disease in tropical Africa and Latin America [142]. YFV vaccine development was a high priority, considering the significant socioeconomic burden of the disease. In 1937, Max Theiler and colleagues produced a YFV live-attenuated vaccine, the 17D vaccine strain, through 176th passage in sub-cultures of wild-type YFV Asibi strain [143,144]. In the subsequent near-century of clinical use, the 17D strain remains an example of safety and efficacy, providing long-term protection after a single dose.

Despite the availability of the effective YF17D vaccine, YFV re-emergence continues to be problematic. The number of YF cases increased over the past two decades due to rapid urbanization, climate change, deforestation, poor vector controls, poor vaccine coverage in high-risk areas, and global vaccine stockpile shortages. Alternative strategies to improve the production, transportation, and storage are required to accommodate the increasing demand for YF17D vaccine and to achieve the WHO EYE (Eliminate Yellow fever Epidemics) goal [145].

One of these promising technologies is an iDNA plasmid encoding the RNA genome of YFV 17D vaccine downstream from a CMV promoter [27] (Figure 1C). This vaccine was engineered to transcribe the full-length viral RNA and to launch the 17D vaccine virus in vitro and in vivo. The YFV cDNA is notoriously toxic in *E. coli*, and the first infectious clone for YFV was assembled in vitro [146]. Initially, iDNA plasmids containing full-length YFV 17D cDNA produced low yields of DNA in *E. coli*, likely due to potentially toxic products expressed in bacterial cells. To address plasmid manufacturing in *E. coli*, intron sequences were cloned into the YFV 17D cDNA to disrupt the potentially toxic ORF, as described above for JEV. Transfection with 10 ng of YFV 17D iDNA plasmid was sufficient to start replication of iDNA 17D vaccine virus in vitro (Table 2). Safety of the iDNA-derived 17D virus was confirmed in AG129 mice deficient in IFN-α/β/γ receptors. Both viruses, classical 17D and iDNA-derived 17D, had similar replication kinetics in brain (neurotropism) and in liver (viscerotropism) tissues. However, replication of iDNA-derived 17D in the liver was less active compared to classical 17D [27]. Vaccination of BALB/c mice with a single 20 μg dose of iDNA YF17D plasmid resulted in seroconversion and elicitation of virus-specific neutralizing antibodies, correlates of protection (Table 2). Neutralizing antibody titers were equivalent with, or exceeded, those in control mice vaccinated with parental YF17D [27].

Another approach to preparing a DNA-launched YFV vaccine involved the bacterial artificial chromosome (BAC) [45]. Similar to the earlier study [27], a plasmid with the full-length YFV 17D cDNA was found genetically unstable during amplification in *E. coli*. Therefore, a low-copy number BAC clone pBeloBAC-FLYF with reduced toxicity was constructed and studied in cell culture and in mice. High titers of YFV-17D were observed upon transfection of the BAC DNA into cells, whereas a mutant containing deletion in the capsid-coding region, pBeloBAC-YF/ΔC, was restricted to a single round of infection, with no detectable release of progeny virions. Next, the homologous prime–boost immunization of AAD transgenic mice (with a humanized MHC-I gene) with both pBeloBAC-FLYF and pBeloBAC-YF/ΔC elicited specific and dose-dependent cellular immune responses to YFV 17D. Finally, vaccination of A129 mice with pBeloBAC-FLYF resulted in the induction of YFV-specific neutralizing antibodies in vaccinated animals. These studies showed that DNA-launched live-attenuated virus immunization can be another vaccination strategy for YFV [27,45].

## 6. Challenges and Perspectives for iDNA Vaccines

Traditionally, the rescue of full-length infectious cDNA clones included a transcription strategy based on the in vitro transcription of viral RNA using T7 or SP6 bacteriophage RNA polymerase. The full-length RNA is then transfected into cultured permissive cells to produce live virus that can be harvested for vaccine preparation. In contrast, the iDNA-launched approach is based on intracellular transcription of viral genomic RNA from eukaryotic promoters in transfected cells and does not require in vitro transcription. Therefore, iDNA technology relies upon direct transfection of DNA into mammalian cells to generate virus (Figure 1).

Challenges for iDNA vaccines include the need to design the plasmid for optimal intracellular transcription. The cell can be a hostile environment to foreign DNA and RNA [53,147], and can potentially affect DNA plasmid integrity and transport DNA to the nucleus, as well as synthesize viral RNA in the nucleus, and transport intact RNA to the cytoplasm. Alphaviruses and flaviviruses replicate in the cytoplasm. However, iDNA transcription of the full-length infectious RNA genome occurs in the nucleus and precedes viral RNA transport to the cytoplasm. This opens the possibility for the degradation of the full-length infectious genomic RNA in the nucleus by nucleases or splicing via cryptic splice sites. Additional potential problems could include RNA transport from the nucleus through the nuclear pore to the cytoplasm, as well as levels of gene expression. Once the RNA exits the nucleus, the RNA functions as infectious viral RNA and the live-attenuated vaccine virus is launched. This results in the induction of immune responses that subsequently clear the viral infection. However, the details and the longevity of genomic RNA production and gene expression from the DNA constructs remains to be studied.

Another difficulty is that full-length cDNA clones are sometimes unstable in *E. coli*. For example, the challenges in preparation of the full-length YF cDNA are well known [27,45,146]. Preparation of the full-length clones is often hampered by the instability of plasmids due to a transcriptional activity of cryptic promoters in *E. coli*, resulting in synthesis of potentially toxic products for the bacterial host. Strategies have been implemented to circumvent this challenge. One strategy involves the cloning of introns capable of disrupting potentially toxic ORFs [27,67]. Another strategy includes the synthesis of BAC clones with lower copy numbers and lower toxic effects, resulting in improved stability. Synthetic methods to prepare DNA with predesigned features can potentially allow screening and removal of the putative *E. coli* promoters and cryptic splice sites.

The accessibility of a prototype live-attenuated virus as the parent for the development of the full-length iDNA clone can present an additional challenge. For CHIKV and VEEV alphaviruses, live-attenuated vaccines were previously developed, characterized, and evaluated in early clinical trials [92,114]. Likewise, live-attenuated viral isolates with human clinical history are known for JEV and YFV flaviviruses. However, for other viruses, including many alphaviruses and flaviviruses, there are no clinically tested, or FDA approved, counterpart viruses. Part of the reason is that the preparation of traditional live-attenuated vaccines using multiple passages in cell culture or in vivo is a time-consuming and complex process with no guarantee of success. In addition, in the regulatory environment, empirically attenuated vaccines have significant challenges for approval. Advanced knowledge in the molecular biology of RNA viruses and currently available molecular techniques, including reverse genetics and bioinformatics, provide a powerful tool for the rational design and development of live-attenuated vaccines. Successful rational design and generic mutations are needed for the generation of recombinant iDNA clones to launch replication of attenuated viruses after transfection in vitro or in vivo. Such generic attenuating mutations involving duplication of the 26S promoter, insertion of an IRES element, deletions, or the rearrangement of structural genes of the alphavirus DNA-launched vaccines have been used to develop candidate vaccines [25,26,97,127].

Furthermore, iDNA vaccines for novel viruses can be developed by using a vectored approach in which the iDNA of the vector virus is modified to include the gene(s) of the virus of interest. For example, aplasmid expressing YF 17D vaccine was configured to express the reporter gene [57], and candidate dengue vaccines were prepared by replacing YF structural genes with the corresponding dengue genes in the YF genome [148].

## 7. Discussion and Conclusions

Single-dose vaccines for epidemic-relevant pathogenic alpha- and flaviviruses are needed to prevent infections and respond to increasing reports of disease outbreaks and epidemics. In recent years, progress was reported in the preparation and evaluation of immunogenic, DNA-launched live-attenuated virus vaccines. The safety, immunogenicity, and efficacy of these vaccines were documented, providing a foundation for clinical development. DNA-launched live-attenuated experimental vaccines were described for epidemic-relevant alpha-s and flaviviruses including CHIKV, VEEV, WNV, JEV, and YFV. Nearly all human populations on the planet, except those in the arctic regions, are at risk of infection with at least one of these viruses. Recent changes in climate, urbanization, commerce, and migration favor the spread of these viruses from endemic regions to new areas, which is exemplified by the introduction of WNV to New York in 1999, and the introduction of CHIKV to the Americas in 2013.

Traditionally, the advantage of DNA vaccines is their remarkable genetic and chemical stability, while live-attenuated virus vaccines have the advantage of inducing rapid, long-term, highly protective immunity after a single-dose vaccination. The DNA-launched vaccine approach combines these by launching a live-attenuated vaccine in vivo from iDNA, without the need for external cell substrates or multiple virus passages for vaccine production. As a result of “manufacturing” live-attenuated iDNA vaccines in vivo, these vaccine viruses are expected to have a more tightly characterized population than in traditional live-attenuated vaccines, which minimizes reversion mutations and represents a safety advantage. In addition to enhanced genetic stability, the iDNA technology also has favorable chemical and thermal stabilization, avoiding cold chain requirements. In addition, needle-free high-density microarray patch technology seems to be feasible in the very near feature for both mRNA and DNA vaccines. Because of these features, DNA-launched virus technology may have advantages for epidemic/pandemic preparedness. Potentially, iDNA vaccine technology can also facilitate preparation of molecular repositories of existing viral vaccines. Essentially, the DNA-launched vaccine platform allows effective transformation of live-attenuated alpha- and flavivirus vaccines into the format of DNA vaccine, as well as facilitating the preparation of improved vaccines using local manufacturing, rational design, and reverse genetics. Reverse genetics can also be applied to the generation of DNA-launched vaccines containing reporter molecules that have applications to the development of advanced animal models, allowing further refinement in the preclinical study of the safety and efficacy of iDNA vaccines [149]. Recent advances in DNA-launched viral vaccines warrant further research on this technology [25,26,27,45,46,66].

However, additional studies are needed on DNA-launched vaccines to determine the longevity of virus expression from DNA, and to determine the immunological mechanisms of protective responses such as activation of innate immunity and the long-term immune memory responses.

Vaccines are critically important to protect populations against emerging pathogens [47,150]. Due to recent increases in outbreaks of emerging viral diseases, multiple vaccine platforms are being developed to prepare preventive countermeasures, including recombinant platforms such as mRNA and DNA vaccines [17], live-attenuated viruses [91,151], viral vectors [120,121,122,152,153] and virus-like particles [10,154,155,156].

Future development of DNA-launched vaccines is expected to include several aspects. First, clinical trials are needed to confirm the feasibility of an iDNA approach in humans. Alphavirus and flavivirus infections represent an acute and growing threat in many parts of the world. For example, CHIKV and ZIKV have been considered, along with two members of the filovirus family, Ebolaviruses and Marburgviruses, as being among the top priority pathogens for vaccine development [150]. WNV is another priority pathogen for vaccine development [150].

Second, preclinical research is needed to determine the mechanisms of DNA-launched vaccines, such as the longevity of RNA and virus expression from iDNA, the role of innate, humoral, and cell-mediated immune responses, as well as long-term immune memory. Preliminary data demonstrated advantages in the safety, immunogenicity, and efficacy of this platform, including the use of lower iDNA doses to achieve protection and enhanced genetic stability compared to traditional live-attenuated vaccines [25,26,46]; however, additional research is needed.

Finally, additional alpha- and flaviviruses, as well as other viruses, can be potentially configured into the DNA-launched vaccine format. Reverse genetics tools applied to the rational design of iDNA vaccines provides an effective route to develop countermeasures for Western and Eastern equine encephalitis alphaviruses, as well as for multiple flaviviruses, including tick-borne encephalitis, dengue, and Zika viruses. Considering the available preliminary data on iDNA-launched, live-attenuated virus vaccines, this novel technology is positioned for further development.

## Figures and Tables

**Table 1:** Comparison of live-attenuated vaccines, mRNA, traditional DNA vaccines, and iDNA vaccines.

Vaccine Feature	Live-Attenuated Virus	mRNA	Traditional DNA	iDNA
Vaccine Formula	Live Virus	mRNA	Plasmid DNA	Plasmid DNA
Vaccine Antigen	Live Virus	Subunit	Subunit	Live Virus
Genetic stability		+	+	+
Simple production		+	+	+
High purity		+	+	+
No cold chain			+	+
Single-dose vaccine	+			+
Rapid protection	+			+

**Table 2:** Examples of experimental iDNA vaccines.

Virus Family	Vaccine	Dose, In Vitro	Dose, In Vivo	Vaccine Route	Viremia	Neutralizing Antibody	Protection	References
Alpha	CHIKV	5 ng	10 μg	IM/EP	+/−	+ (10/10)	+ (10/10)	[25]
	VEEV	8 ng	50 μg	IM/EP	+/−	+ (10/10)	+ (10/10)	[26]
			10 μg	IV	+/−		+	
			20 μg	TD	−	+	nt	[64]
Flavi	WNV		0.1–10 μg	IM	+	+	+	[28,30,65,66]
	JEV	2 μg	nt	n/a	n/a	n/a	n/a	[29,67]
	YFV	10 ng	20 μg	IM/EP	−	+ (10/10)	nt	[27]
		5 μg	0.1–10 μg	IM/EP	+	+	nt	[45]

CHIKV, Chikungunya virus; VEEV, Venezuelan equine encephalitis virus; WNV, West Nile virus; JEV, Japanese encephalitis virus; YFV, yellow fever virus; IM, intramuscular; IM/EP, intramuscular/electroporation; IV, intravenous; TD, transdermal (hollow microneedles); nt, not tested; n/a, not applicable. Adapted from [24].
